# Activation of proteinase-activated receptor 2 in human osteoarthritic cartilage upregulates catabolic and proinflammatory pathways capable of inducing cartilage degradation: a basic science study

**DOI:** 10.1186/ar2329

**Published:** 2007-11-21

**Authors:** Christelle Boileau, Nathalie Amiable, Johanne Martel-Pelletier, Hassan Fahmi, Nicolas Duval, Jean-Pierre Pelletier

**Affiliations:** 1 Osteoarthritis Research Unit, University of Montreal Hospital Centre, Notre-Dame Hospital, 1560 Sherbrooke Street East, Montreal, Quebec, H2L 4M1, Canada; 2 Pavillon des Charmilles, 1487, boul. des Laurentides, Vimont, Quebec, H7M 2Y3, Canada

## Abstract

Proteinase-activated receptors (PARs) belong to a family of G protein-coupled receptors. PARs are activated by a serine-dependent cleavage generating a tethered activating ligand. PAR-2 was shown to be involved in inflammatory pathways. We investigated the *in situ *levels and modulation of PAR-2 in human normal and osteoarthritis (OA) cartilage/chondrocytes. Furthermore, we evaluated the role of PAR-2 on the synthesis of the major catabolic factors in OA cartilage, including metalloproteinase (MMP)-1 and MMP-13 and the inflammatory mediator cyclooxygenase 2 (COX-2), as well as the PAR-2-activated signalling pathways in OA chondrocytes. PAR-2 expression was determined using real-time reverse transcription-polymerase chain reaction and protein levels by immunohistochemistry in normal and OA cartilage. Protein modulation was investigated in OA cartilage explants treated with a specific PAR-2-activating peptide (PAR-2-AP), SLIGKV-NH_2 _(1 to 400 μM), interleukin 1 beta (IL-1β) (100 pg/mL), tumor necrosis factor-alpha (TNF-α) (5 ng/mL), transforming growth factor-beta-1 (TGF-β1) (10 ng/mL), or the signalling pathway inhibitors of p38 (SB202190), MEK1/2 (mitogen-activated protein kinase kinase) (PD98059), and nuclear factor-kappa B (NF-κB) (SN50), and PAR-2 levels were determined by immunohistochemistry. Signalling pathways were analyzed on OA chondrocytes by Western blot using specific phospho-antibodies against extracellular signal-regulated kinase 1/2 (Erk1/2), p38, JNK (c-jun *N*-terminal kinase), and NF-κB in the presence or absence of the PAR-2-AP and/or IL-1β. PAR-2-induced MMP and COX-2 levels in cartilage were determined by immunohistochemistry. PAR-2 is produced by human chondrocytes and is significantly upregulated in OA compared with normal chondrocytes (*p *< 0.04 and *p *< 0.03, respectively). The receptor levels were significantly upregulated by IL-1β (*p *< 0.006) and TNF-α (*p *< 0.002) as well as by the PAR-2-AP at 10, 100, and 400 μM (*p *< 0.02) and were downregulated by the inhibition of p38. After 48 hours of incubation, PAR-2 activation significantly induced MMP-1 and COX-2 starting at 10 μM (both *p *< 0.005) and MMP-13 at 100 μM (*p *< 0.02) as well as the phosphorylation of Erk1/2 and p38 within 5 minutes of incubation (*p *< 0.03). Though not statistically significant, IL-1β produced an additional effect on the activation of Erk1/2 and p38. This study documents, for the first time, functional consequences of PAR-2 activation in human OA cartilage, identifies p38 as the major signalling pathway regulating its synthesis, and demonstrates that specific PAR-2 activation induces Erk1/2 and p38 in OA chondrocytes. These results suggest PAR-2 as a potential new therapeutic target for the treatment of OA.

## Introduction

Osteoarthritis (OA) can be defined as a complex degradative and repair process in cartilage, subchondral bone, and synovial membrane. The factors responsible for the appearance and progression of joint structural changes in OA have been the subject of intensive research for a few decades. Although significant progress has been made in the understanding of the pathophysiological pathways responsible for some of the changes, much remains to be done to establish a therapeutic intervention that can effectively reduce or stop the progression of the disease.

OA is characterized mainly by degradation of the cartilage. The alterations in OA cartilage are numerous and involve morphologic and synthetic changes in chondrocytes as well as biochemical and structural alterations in the extracellular matrix macromolecules [1]. In OA, the chondrocytes are the first source of enzymes responsible for cartilage matrix catabolism, and it is widely accepted that the metalloproteinase (MMP) family has a major involvement in the disease process [2]. Moreover, considerable evidence has accumulated indicating that the proinflammatory cytokines synthesized and released by chondrocytes and synovial membrane are crucial in OA cartilage catabolic processes and have an important impact in the development/progression of the disease [1].

In addition to cytokines, other mediators could play a major role in the OA pathological process. A member of the newly identified cell membrane receptor family, the proteinase-activated receptors (PARs), has been shown to be involved in inflammatory pathways. These receptors belong to a novel family of seven-transmembrane G protein-coupled receptors that are activated through a unique process. The cleavage by serine proteases of the PAR *N*-terminal domains unmasks a new *N*-terminal sequence that acts as a tethered ligand, binding and activating the receptor itself [3,4]. This activation is an irreversible phenomenon: the cleaved receptor is activated, internalized, and degraded. The cell membrane PARs are restored from the intracellular pool [5].

This receptor family consists of four members, PAR-1 to PAR-4. PAR-1, PAR-3, and PAR-4 are activated by thrombin, whereas PAR-2 is activated mainly by trypsin but also by mast cell tryptase. PARs are expressed by several cell types, including platelets and endothelial and inflammatory cells, and are implicated in numerous physiological and pathological processes [3,4]. PAR-2 has also been found to be involved in multiple cellular responses related to hyperalgesia. For example, Kawabata and colleagues [6] showed that the PAR-2 activation by a specific agonist elicited thermal hyperalgesia and nociceptive behavior, and Vergnolle and colleagues [7] demonstrated that the thermal and mechanical hyperalgesia were reduced in PAR-2-deficient mice. In addition, PAR-2 is implicated in neurogenic inflammation [8] as well as inflammatory conditions, including those seen in rheumatoid arthritis [9]. In that regard, an important role for PAR-2 in the mouse adjuvant-induced arthritis model has been shown by using a *PAR-2 *gene knockout mouse in which the appearance of inflammation was significantly delayed [10,11]. Recently, PAR-2 expression has been found in chondrocytes and synovial fibroblasts [12,13].

This study aimed to investigate the *in situ *levels and modulation of PAR-2 in human normal and OA cartilage, determine its functional consequences on this tissue, and evaluate the chondrocyte signalling pathways involved in PAR-2 activity. We showed that PAR-2 is present at increased levels in human OA cartilage and that its level is modulated by the proinflammatory cytokines interleukin 1 beta (IL-1β) and tumor necrosis factor-alpha (TNF-α). Specific PAR-2 activation stimulates major pathophysiological pathways involved in the OA process, including MMP-1 and MMP-13 as well as cyclooxygenase 2 (COX-2), through the activation of extracellular signal-regulated kinase 1/2 (Erk1/2) and p38.

## Materials and methods

### Specimen selection

Human articular cartilage was obtained from femoral condyles or tibial plateaus. Normal knees were obtained within 12 hours of death (mean age ± standard deviation [SD]: 52 ± 14 years). The cartilage was examined macroscopically and microscopically to ensure that only normal tissue was used. Human OA specimens were from patients undergoing total knee arthroplasty (mean age ± SD: 76 ± 5 years). All patients with OA were evaluated by a certified rheumatologist and were diagnosed as having OA based on the criteria developed by the American College of Rheumatology Diagnostic Subcommittee for OA [14]. These specimens represented moderate to severe OA as defined according to macroscopic criteria. This project and the informed consent form were approved by the institutional Ethics Committee Board of the University of Montreal Hospital Centre.

### Cartilage explant culture

Normal and OA cartilage explants (approximately 150 mg) were dissected and fixed in TissuFix #2 (Chaptec, Montreal, QC, Canada) and processed directly after acquisition from the donor for immunohistochemistry (basal synthesis) or incubated in Dulbecco's modified Eagle's medium (DMEM) supplemented with 10% heat-inactivated fetal calf serum (FCS) and an antibiotics mixture (100 units/mL of penicillin base and 100 μg/mL of streptomycin base) (Gibco-BRL Life Technologies, now part of Invitrogen Corporation, Burlington, ON, Canada) at 37°C in a humidified atmosphere of 5% CO_2_/95% air. The conditions used were optimal for cartilage explant cultures. Cartilage explants were treated for 48 hours by IL-1β (100 pg/mL), TNF-α (5 ng/mL), and transforming growth factor-beta-1 (TGF-β1) (10 ng/mL) (all from R&D Systems, Inc., Minneapolis, MN, USA) or by the synthetic PAR-2-activating peptide (PAR-2-AP), SLIGKV-NH_2 _(0 to 400 μM) (Bachem California, Inc., Torrance, CA, USA), p38 inhibitor (SB 202190 at 10 μM) (Tocris Bioscience, Ellisville, MO, USA), and mitogen-activated protein (MAP) kinase kinase (MEK1/2) inhibitor (PD98059 at 10 μM) and nuclear factor-kappa B (NF-κB) inhibitor (SN50 at 50 μg/mL) (both from EMD Biosciences, Inc., San Diego, CA, USA). Cartilage explants were then processed for PAR-2 immunohistochemistry as described below.

### Chondrocyte culture and treatment

Chondrocytes were released from full-thickness strips of cartilage followed by sequential enzymatic digestion at 37°C, as previously described [15]. Cells were seeded at high density (10^5 ^cells/cm^2^) in tissue culture flasks and were cultured to confluence in DMEM supplemented with 10% FCS and an antibiotics mixture (Invitrogen Corporation) at 37°C in a humidified atmosphere. To ensure phenotype, only first-passage cultured chondrocytes were used.

The chondrocytes were harvested with Cell Dissociation Buffer (Invitrogen Corporation) (which contains no protease), seeded, and cultured in DMEM containing 10% FCS at 37°C until confluence. Cells were further incubated with DMEM containing 2.5% FCS and treated with IL-1β (100 pg/mL), TNF-α (5 ng/mL), and TGF-β1 (10 ng/mL) (all from R&D Systems, Inc.) and the synthetic PAR-2-AP, SLIGKV-NH_2 _(0 to 400 μM) (Bachem California, Inc.), for 72 hours for PAR-2 protein determination and 0 to 60 minutes for signalling pathways.

### RNA extraction, reverse transcription, and real-time polymerase chain reaction

Total RNA was extracted from chondrocytes as previously described [15]. Briefly, total RNA was extracted with TRIzol^® ^according to the manufacturer's instructions (Invitrogen Corporation), and genomic DNA was removed following the manufacturer's instructions (Ambion, Inc., Austin, TX, USA). The RNA was quantified with the RiboGreen^® ^RNA quantification kit (Molecular Probes Inc., now part of Invitrogen Corporation). cDNA was reverse-transcribed from 1 μg of total RNA purified in a 50-μL reaction mixture containing 1 mM each of deoxynucleotide triphosphates (Invitrogen Corporation), 0.4 U/μL RNase inhibitor and 2.5 μM of random hexamer (both from GE Healthcare, Baie d'Urfé, QC, Canada), 2.5 U/μL of reverse transcriptase (Invitrogen Corporation), 5 mM of MgCl_2_, and 1× of polymerase chain reaction (PCR) buffer. The reaction mixture was incubated in a DNA thermal cycle at 42°C for 15 minutes and then stored at -20°C before use. Real-time PCR was performed using primers specific for the human PAR-2 and for the human housekeeping gene glyceraldehydes-3-phosphate dehydrogenase (*GAPDH*). The primers were 5'-GAAGCCTTATTGGTAAGGTTG (sense) and 5'-CAGAGAGGAGGTCAGCCAAG (anti-sense) for *PAR-2 *and 5'-CAGAACATCATCCCTGCCTCT (sense) and 5'-GCTTGACAAAGTGGTCGTTGAG (anti-sense) for *GAPDH*. In brief, 10 μL of the cDNA obtained from the reverse transcription reactions was amplified in a total volume of 25 μL consisting of 1× Quantitect SYBR Green PCR Master Mix (Qiagen Inc., Mississauga, ON, Canada), 0.5 U/reaction uracil-N-glycosylase (Invitrogen Corporation), and gene-specific primers that were added at a final concentration of 200 nM. Real-time quantitation mRNA was performed in the Rotor-Gene 6^® ^RG-3000A (Corbett Research, Mortlake, NSW, Australia) according to the manufacturer's instructions. Data were processed with Rotor-Gene version 6 software and were given a threshold cycle (C_T_) corresponding to the PCR cycle at which an increase in reporter fluorescence above a baseline signal can first be detected. Plasmid DNAs containing target gene sequences were used to generate the standard curves. The C_T _was converted to number of molecules, and the values for each sample were calculated as the ratio of the number of molecules of the target gene to the number of molecules of *GAPDH *and were expressed as arbitrary units.

### Immunohistochemistry

Cartilage specimens were processed for immunohistochemical analysis as previously described [16]. Briefly, specimens were fixed in TissuFix #2 for 24 hours and then embedded in paraffin. Sections (5 μm) of paraffin-embedded specimens were placed on Superfrost Plus slides (Fisher Scientific, Nepean, ON, Canada), deparaffinized in toluene, rehydrated in a reverse-graded series of ethanol, and preincubated with chondroitinase ABC 0.25 units/mL (Sigma-Aldrich, Oakville, ON, Canada) in phosphate-buffered saline (PBS) (pH 8.0) for 60 minutes at 37°C. Subsequently, the specimens were washed in PBS, incubated in 0.3% Triton X-100 for 20 minutes, and placed in 3% hydrogen peroxide/PBS for 15 minutes. Slides were further incubated with a blocking serum (Vectastain ABC assay; Vector Laboratories, Burlingame, CA, USA) for 60 minutes, after which they were blotted and then overlaid with the primary antibody against mouse anti-human PAR-2 (1:50; Zymed Laboratories Inc., now part of Invitrogen Corporation), mouse anti-human COX-2 (1:25; Cedarlane Laboratories Ltd., Burlington, ON, Canada), mouse anti-human MMP-1 (1:40; EMD Biosciences, Inc.), and goat anti-human MMP-13 (1:6; R&D Systems, Inc.) for 18 hours at 4°C. Each slide was washed three times in PBS (pH 7.4) and incubated with the second antibody (anti-mouse or anti-goat; Vector Laboratories) for 1 hour at room temperature, followed by a staining with the avidin-biotin-peroxidase complex method (Vectastain ABC assay). The color was developed with 3,3'-diaminobenzidine (DAKO Diagnostics Inc., Mississauga, ON, Canada) containing hydrogen peroxide. Slides were counterstained with eosin. All incubations were carried out in a humidified chamber. Each section was examined under a light microscope (Leitz Orthoplan; Leica Inc., St. Laurent, QC, Canada). Two control procedures were performed according to the same experimental protocol: (a) omission of the primary antibody and (b) substitution of the primary antibody with an autologous preimmune serum. Controls showed only background staining.

Positive cells were quantified as previously described [17]. In brief, three sections for each specimen were examined (×40; Leica Orthoplan) from the cartilage superficial zone (which included the superficial and upper intermediate layers). The sections were scored, and the resulting data were integrated as a mean for each specimen. The total numbers of chondrocytes and of those staining positive for the specific antigen were determined. The final results were expressed as the percentage of chondrocytes staining positive for the antigen (cell score), with the maximum score being 100%. Each slide was examined by two independent readers.

### Western blot

Total proteins were extracted with 0.5% sodium dodecyl sulfate (SDS) (Invitrogen Corporation) supplemented with protease inhibitors. The protein level was determined using the bicinchoninic acid protein assay, and 10 μg of the protein was electrophoresed on a discontinuous 4% to 12% SDS gel polyacrylamide. The proteins were transferred electrophoretically onto a nitrocellulose membrane (Bio-Rad Laboratories Ltd., Mississauga, ON, Canada) for 1 hour at 4°C. The efficiency of transfer was controlled by a brief staining of the membrane with Ponceau Red and destained in water and TTBS 1× (Tris 20 mM, NaCl 150 mM [pH 7.5], and 0.1% Tween 20) before immunoblotting.

The membranes were incubated overnight at 4°C with 5% skimmed milk in SuperBlock Blocking Buffer-Blotting in Tris-buffered saline (Pierce, Rockford, IL, USA) or in TTBS 1× only. The membranes were then washed once with TTBS 1× for 10 minutes and incubated in SuperBlock Blocking Buffer-Blotting and TTBS 1× (SuperBlock 1:10 with TTBS 1×) or in TTBS 1× (for PAR-2 antibody only) with 0.5% skimmed milk supplemented with the mouse anti-human PAR-2 (1:1,000; Invitrogen Corporation) and with mouse anti-human antibodies against the phosphorylated forms of p38 (1:1,000), Erk1/2 (1:5,000), c-jun *N*-terminal kinase (JNK) (1:5,000), and NF-κB (p65) (1:5,000) (all from New England Biolabs Ltd., Pickering, ON, Canada) overnight at 4°C. The membranes were washed with TTBS 1× and incubated for 1 hour at room temperature with the second antibody (1:20,000; anti-mouse immunoglobulin G horseradish peroxidase-conjugated; Pierce) and washed again with TTBS 1×. Detection was performed by chemiluminescence using the Super Signal^® ^ULTRA chemiluminescent substrate (Pierce) and exposure to Kodak Biomax photographic film (GE Healthcare). The band intensity was measured by densitometry using TotalLab TL100 Software (Nonlinear Dynamics Ltd., Newcastle upon Tyne, UK), and data were expressed as arbitrary units, in which the control was assigned a value of 100%.

### Statistical analysis

Values are expressed as median (range) or as mean ± standard error of the mean (SEM) when appropriate. Statistical analysis was performed using the Mann-Whitney *U *test.

## Results

### PAR-2 expression and synthesis

**Figure 1:**
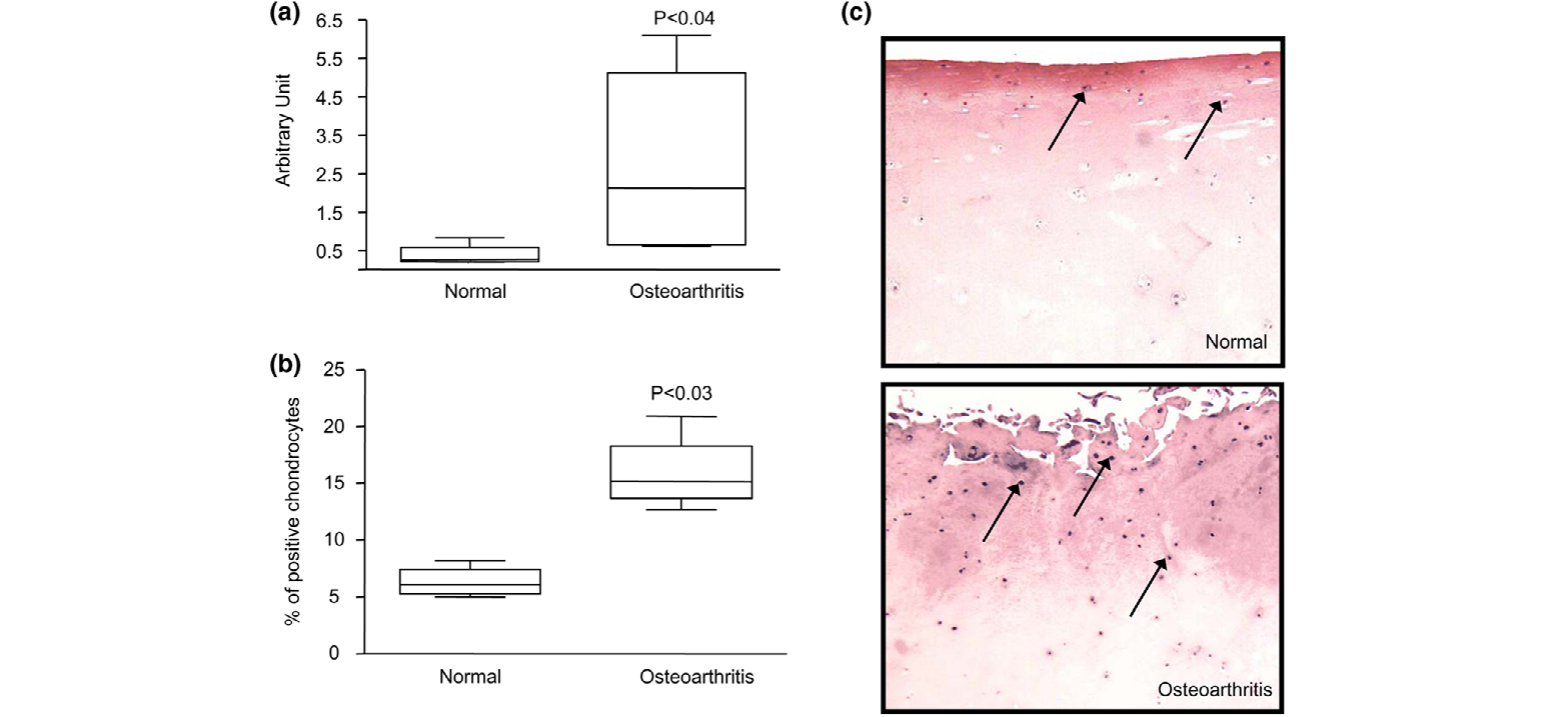
Proteinase-activated receptor 2 (PAR-2) gene expression and protein synthesis. **(a) **mRNA levels, as determined by real-time quantitative polymerase chain reaction as described in Materials and methods, in normal (*n *= 4) and osteoarthritis (*n *= 6) chondrocytes. **(b) **PAR-2 immunostaining in normal (*n *= 4) and osteoarthritis (*n *= 4) cartilage. The percentage of positive chondrocytes represents the number of chondrocytes staining positive for PAR-2 of the total number of chondrocytes. Data are expressed as median and range and are presented as box plots, in which the boxes represent the first and third quartiles, the line within the box represents the median, and the lines outside the box represent the spread of values. *P *values indicate the comparison of normal to osteoarthritis cartilage using the Mann-Whitney *U *test. **(c) **Representative sections showing PAR-2 immunostaining in normal and osteoarthritis cartilage. The arrows refer to positive chondrocytes.

The levels of *PAR-2 *mRNA in normal (*n *= 4) and OA (*n *= 6) chondrocytes were determined by real-time PCR. As illustrated at Figure 1a, *PAR-2 *showed a significantly higher level (mean increase of 6.5-fold; *p *< 0.04) in OA chondrocytes than in normal chondrocytes. Similarly, PAR-2 protein levels, detected by immunohistochemistry, were significantly upregulated in OA (*n *= 4) compared with normal (*n *= 4) cartilage (mean increase of 2.5-fold; *p *< 0.03) (Figure 1b). Figure 1c illustrates that PAR-2 is localized mainly in the superficial zone (consisting of the superficial and upper intermediate layers) in normal and OA cartilage.

### Regulation of PAR-2 synthesis

**Figure 2:**
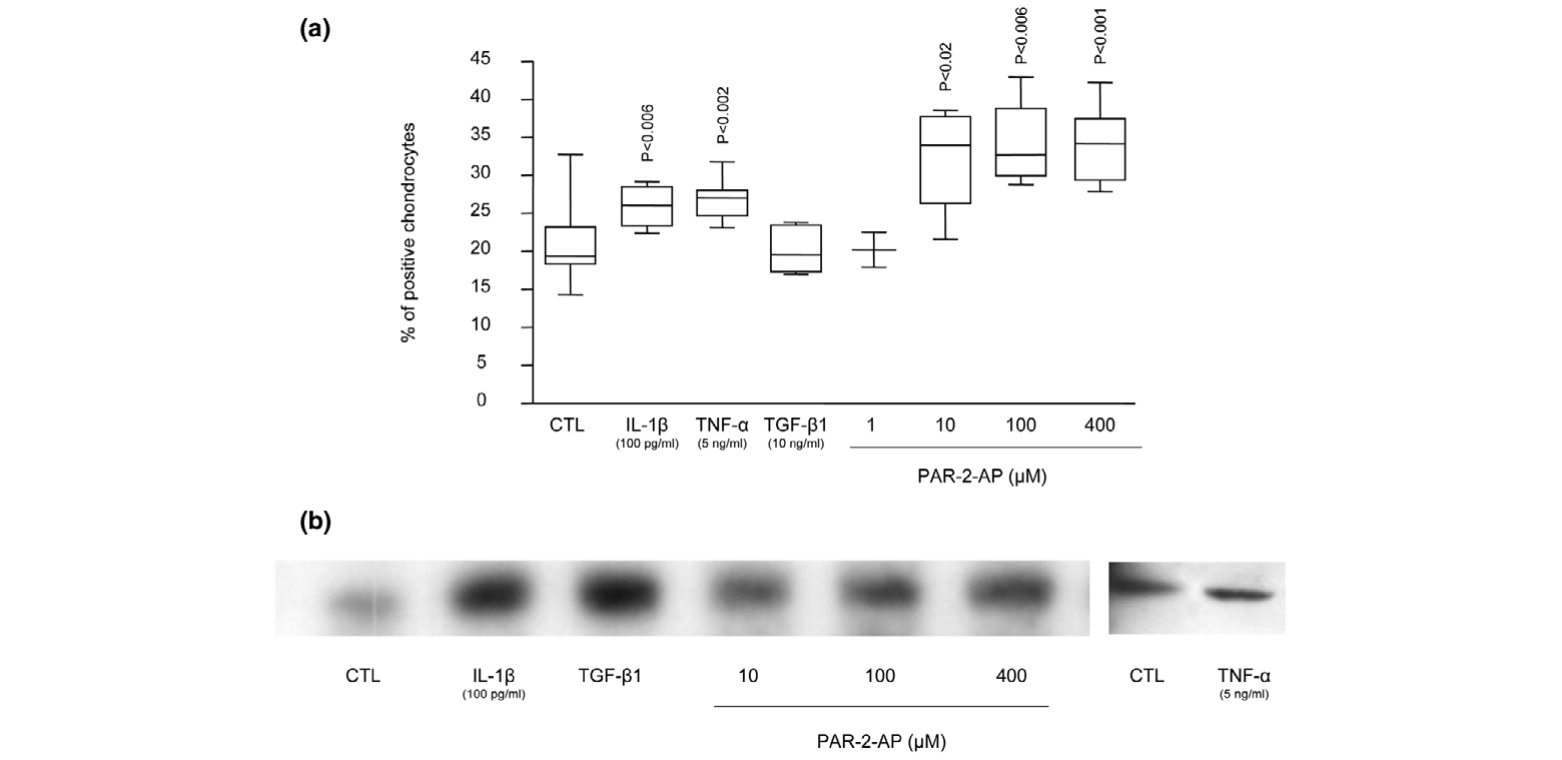
Proteinase-activated receptor 2 (PAR-2) synthesis regulation. **(a) **PAR-2 immunostaining in osteoarthritis (OA) cartilage explants untreated (*n *= 16) and treated with interleukin 1 beta (IL-1β) (*n *= 9), tumor necrosis factor-alpha (TNF-α) (*n *= 9), transforming growth factor-beta-1 (TGF-β1) (*n *= 5), PAR-2-activating peptide (PAR-2-AP) 1 μM (*n *= 3), PAR-2-AP 10 μM (*n *= 4), PAR-2-AP 100 μM (*n *= 4), and PAR-2-AP 400 μM (*n *= 8) for 48 hours in Dulbecco's modified Eagle's medium (DMEM) 10% fetal calf serum (FCS). **(b) **Representative Western blot of PAR-2 synthesis in OA monolayer chondrocytes (*n *= 3) incubated for 72 hours in DMEM 2.5% FCS in the absence (CTL) or presence of IL-1β, TGF-β1, PAR-2-AP 10 μM, PAR-2-AP 100 μM, and PAR-2-AP 400 μM. *P *values indicate the comparison with the untreated (CTL) specimens.

To explore the mechanism underlying PAR-2 modulation in human OA cartilage, we studied the effects of two proinflammatory cytokines, IL-1β and TNF-α, the growth factor TGF-β1, and a specific PAR-2 activator agonist, the PAR-2-AP, on cartilage explant cultures. Data showed that, in OA cartilage, PAR-2 levels were significantly upregulated by IL-1β (*n *= 9; *p *< 0.006) and TNF-α (*n *= 9; *p *< 0.002) but not by TGF-β1 (*n *= 5) (Figure 2a). The PAR-2-AP (*n *= 4 to 8) also significantly increased the PAR-2 level, starting at 10 μM (*p *< 0.02) (Figure 2a). Interestingly, PAR-2-AP appeared to be more efficient at increasing the level of PAR-2 protein than the cytokines, and mean increases of 53%, 64%, and 63% were found for PAR-2-AP 10, 100, and 400 μM, respectively, compared with 24% for IL-1β and 29% for TNF-α.

On OA monolayer chondrocytes (*n *= 3), data obtained for IL-1β and TNF-α (Figure 2b) were similar to those from OA cartilage explants. However, in contrast to cartilage explants, OA chondrocyte treatment with TGF-β1 (*n *= 3) yielded a marked PAR-2 protein increase (Figure 2b). As Xiang and colleagues [12] reported that TGF-β decreased the level of PAR-2 in OA chondrocytes, we further validated our findings by investigating the effect of TGF-β1 on PAR-2 expression levels on normal (*n *= 2) and OA (*n *= 3) chondrocytes. Data showed that on both sets this factor markedly increased PAR-2 expression levels (20- and 42-fold, respectively; data not shown). In the OA cells as in cartilage, PAR-2-AP treatment (*n *= 3) also markedly increased PAR-2 protein levels (Figure 2b).

**Figure 3:**
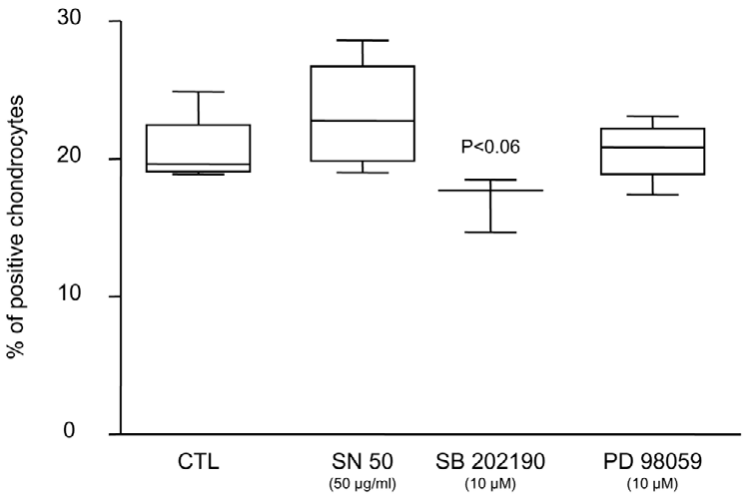
Signalling pathways of proteinase-activated receptor 2 (PAR-2) synthesis. PAR-2 immunostaining in osteoarthritic cartilage untreated (*n *= 4) and treated with SB 202190 (inhibitor of p38; *n *= 3), PD 98059 (inhibitor of MEK1/2; *n *= 4), and SN50 (inhibitor of nuclear factor-kappa B; *n *= 4). *P *value indicates the comparison with the untreated (CTL) specimens. MEK1/2, mitogen-activated protein kinase kinase.

To explore the signalling pathways involved in the regulation of PAR-2 synthesis, human OA cartilage explants were incubated for 48 hours with the MAP kinase inhibitor SB 202190, inhibitor of p38; PD 98059, inhibitor of MEK1/2; and SN50, inhibitor of NF-κB. Data (*n *= 3 to 4) revealed that only the p38 inhibitor markedly downregulated (*p *< 0.06) the PAR-2 production in OA cartilage (Figure 3). Erk1/2 and NF-κB inhibitions had no effect on PAR-2 synthesis.

### PAR-2 activation and functional consequences

**Figure 4:**
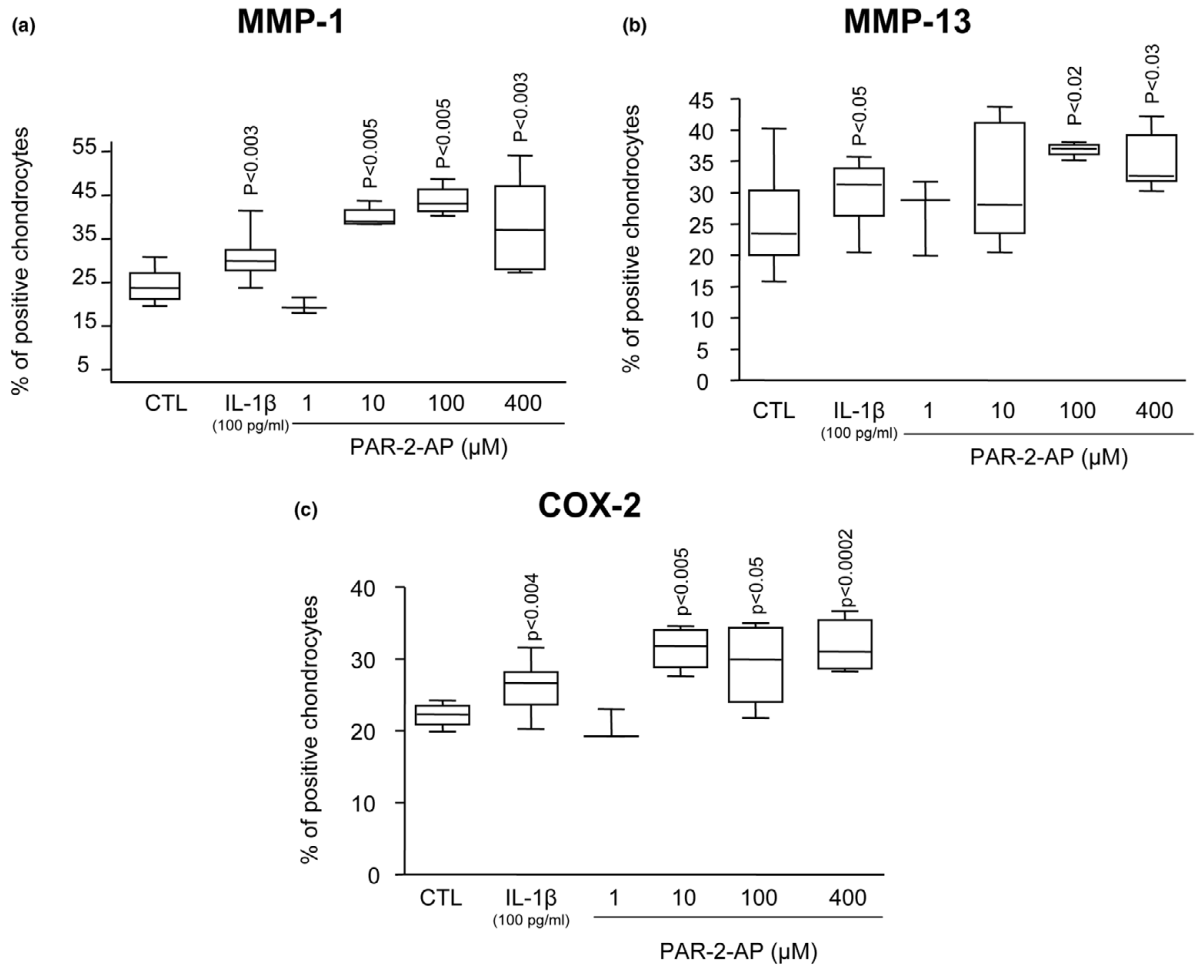
Production of metalloproteinase (MMP)-1 **(a)**, MMP-13 **(b)**, and cyclooxygenase 2 (COX-2) **(c) **following interleukin 1 beta (IL-1β) and specific proteinase-activated receptor 2 (PAR-2) activation. Immunostaining data of osteoarthritic cartilage untreated (*n *= 12) and treated with IL-1β (*n *= 12), PAR-2-activating peptide (PAR-2-AP) 1 μM (*n *= 3), PAR-2-AP 10 μM (*n *= 4), PAR-2-AP 100 μM (*n *= 4), and PAR-2-AP 400 μM (*n *= 9). *P *values indicate the comparison with the untreated (CTL) specimens.

To determine the functional consequence of PAR-2 activation, we studied some of the major catabolic/inflammatory factors involved in OA pathophysiology, including MMP-1, MMP-13, and COX-2 (Figure 4). PAR-2-AP treatment of OA cartilage explants (*n *= 3 to 9) revealed a statistically significant increase starting at concentrations of 10 μM for MMP-1 (*p *< 0.005) and COX-2 (*p *< 0.005) and 100 μM for MMP-13 (*p *< 0.02). For each factor, the increase was localized at the superficial zone. As expected, IL-1β (*n *= 12) showed a statistically significant increase for all of the factors examined. Comparison revealed that this cytokine had a lower induction level on each factor than the PAR-2 activation. IL-1β induced mean increases for MMP-1, MMP-13, and COX-2 of 29%, 20%, and 18% compared with means of 73%, 44%, and 40% for PAR-2-AP.

### PAR-2-induced signalling pathways

**Figure 5:**
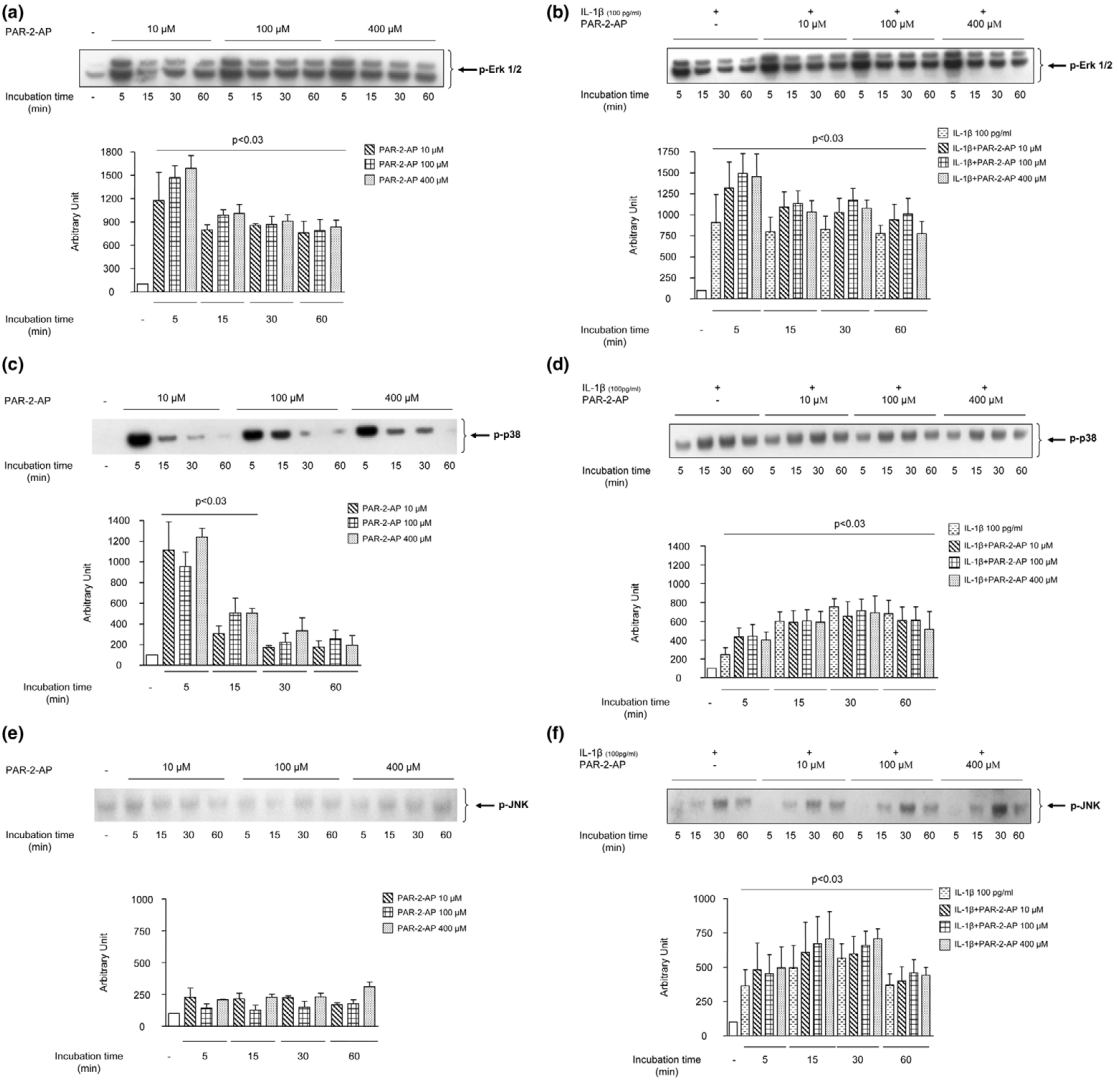
Signalling pathways induced by specific proteinase-activated receptor 2 (PAR-2) activation. Representative Western blot of time and dose curves of PAR-2 signalling in osteoarthritic chondrocytes (*n *= 3 or 4). Phosphorylated forms of Erk1/2 (p-Erk1/2) **(a,b)**, p38 (p-p38) **(c,d)**, and JNK (p-JNK) **(e,f) **treated with PAR-2-activating peptide (PAR-2-AP) at 0 (-), 10, 100, and 400 μM in the absence **(a,c,e) **or presence **(b,d,f) **of interleukin 1 beta (IL-1β) (100 pg/mL) for 0 to 60 minutes **(a,c,e)**. *P *values indicate the comparison between the untreated (-) and the PAR-2-AP-treated chondrocytes. For p-Erk1/2, all of the times and concentrations showed a statistically significant increase (*p *< 0.03). For p-p38, statistical significance was reached for each concentration at 5 and 15 minutes (*p *< 0.03). **(b,d,f) ***P *values indicate the comparison between the untreated and the IL-1β-treated chondrocytes in the absence or presence of PAR-2-AP. Erk1/2, extracellular signal-regulated kinase 1/2; JNK, c-jun N-terminal kinase.

The effect of PAR-2 activation on the phosphorylated levels of three MAP kinases of the OA chondrocytes (*n *= 3 to 4), namely Erk1/2, p38, and JNK, and on NF-κB was analyzed by Western blot using specific antibodies. The activation of PAR-2 in OA chondrocytes using the PAR-2-AP (Figure 5a) yielded within minutes (5 minutes) a sharp phosphorylation of Erk1/2 (p-Erk1/2) (*p *< 0.03). Data also showed that the maximal PAR-2-AP stimulation concentration is 100 μM. At 15 minutes of PAR-2-AP treatment, p-Erk1/2 still showed significantly elevated levels compared with the control (*p *< 0.03) but to a lesser extent than at 5 minutes. PAR-2-AP also induced p38 phosphorylation (p-p38) rapidly, with a maximum stimulation at 5 minutes (*p *< 0.03) (Figure 5c), which declined thereafter but was still statistically significant (*p *< 0.03) at 15 minutes of treatment. No true dose-dependent effect was seen for the transient p38 phosphorylation. The levels of the phosphorylated form of JNK (p-JNK) were very low and were not affected by PAR-2-AP treatment (Figure 5e). Finally, the levels of the phosphorylated form of NF-κB were barely detectable even after treatment with PAR-2-AP (data not shown).

IL-1β significantly (*p *< 0.03) increased the level of the phosphorylated form of each of the MAP kinases studied, with maximums reached at 5 minutes of incubation for p-Erk1/2 and 30 minutes for p-p38 and p-JNK (Figure 5b,d,f). The treatment with PAR-2-AP together with IL-1β yielded an additional effect for p-Erk1/2, but this was not statistically different from the IL-1β alone (Figure 5b). This was also noticed for p38 phosphorylation (Figure 5d) at 5 minutes of incubation. For p-JNK (Figure 5f) and NF-κB (data not shown), the addition of PAR-2-AP did not modify IL-1β-induced activity.

## Discussion

This study is the first to demonstrate that PAR-2 activation in human OA cartilage significantly upregulates the synthesis of important catabolic and proinflammatory mediators involved in the progression of the disease and that the effect is mediated by the activation of Erk1/2 and p38 signalling pathways. Here, we showed that PAR-2 expression and protein levels were significantly increased in OA compared with normal human chondrocytes and that the levels are upregulated by the proinflammatory cytokines IL-1β and TNF-α, an effect previously observed on chondrocytes by Xiang and colleagues [12] and on other cell types [13,18,19]. Our data showing that TGF-β1 on OA chondrocytes, but not on OA cartilage explants, upregulates PAR-2 levels appear contradictory. A possible explanation could be that, in the cartilage explants, large amounts of TGF-β can be entrapped in the extracellular matrix and consequently only a very low concentration of this factor reaches the cells. Indeed, one of the characteristics of some proteoglycans is their interaction with active TGF-β, which provides a tissue reservoir of this factor, thereby modulating its bioavailability [20,21]. Our finding of an increased level of PAR-2 induced by TGF-β1 on OA chondrocytes also differed, to some extent, from the results of Xiang and colleagues [12], who reported a differential effect of TGF-β between normal and OA chondrocytes; TGF-β downregulated PAR-2 levels in OA but increased its levels in normal chondrocytes. A reason for this discrepancy could be that, in the report by Xiang and colleagues, the protein measurement was carried out on a specimen not representative of the heterogeneity of the human sampling; only one normal and one OA chondrocyte were used. Such a possibility has been underlined by the authors [12], who suggest the possible existence of different cell populations such as responders and non-responders.

To explore the mechanism underlying PAR-2 modulation, we used a specific PAR-2 activator agonist, the PAR-2-AP SLIGKV-NH_2_, which corresponds to the first six amino acids of the tethered ligand sequence. This peptide activates PAR-2 independently of proteolytic unmasking of the tethered ligand sequence and triggers G-protein coupling [3]. Our finding that the PAR-2-AP, in addition to activating PAR-2, significantly upregulates the level of the receptor on OA chondrocytes agrees with a recent study showing that another PAR-2-AP agonist, 2-furoyl LIGKV-OH (ASK95), regulates the expression of PAR-2 in umbilical vein endothelial cells [18].

In this study, we also addressed the roles of candidate signalling events able to regulate PAR-2 production and, on the other hand, those responsible for the PAR-2-mediated functional response. These include the major MAP kinases as well as NF-κB. Our results first revealed a major role for p38, but not for MEK1/2 or NF-κB, as regulators of PAR-2 synthesis. These data are important because an essential role for p38 in PAR-2 upregulation could not be applied to all cell types [22]. However, these findings are consistent with our observation that the proinflammatory cytokines IL-1β and TNF-α, which strongly activate p38 in articular joints [23,24], also upregulate PAR-2. Moreover, although the major signalling pathway of TGF-β1 is the Smad system, TGF-β1 was shown to mediate some of its activities (particularly those not related to the growth factor effect) via the p38 pathway [25,26].

No study has yet reported on the PAR-2-mediated functional response in human OA chondrocytes. We demonstrated that both Erk1/2 and p38 pathways, but not those of JNK or NF-κB, are activated very early in response to a specific PAR-2 stimulation. The former signalling pathways, in turn, are widely implicated in the ongoing catabolic events in cartilage degradation. Indeed, Erk1/2 and p38 are the two preferential signalling cascades involved in the production of MMP-1 and MMP-13 by human chondrocytes [27-29] and the p38 activation in COX-2 [30,31].

Interestingly, co-stimulation of chondrocytes with IL-1β and PAR-2-AP showed an additional stimulatory effect, particularly on Erk1/2. A possible explanation is that Erk1/2 is not the preferential pathway mediating the effects of IL-1β; consequently, stimulation by this cytokine may not have reached maximal activation of this pathway. This finding thus indicates that, during the disease process, both PAR-2 and IL-1β could act in cooperation at inducing a catabolic cellular response.

The increased level of PAR-2 in OA compared with normal chondrocytes may be related, in addition to the stimulatory effect of the cytokines, to an increased level of serine proteases in OA cartilage. Indeed, according to the literature, this enzyme family appears to be responsible for the PAR-2 activation [3,4]. In OA cartilage, one of the most important serine protease systems is the plasminogen activator (PA) plasmin, in which the urokinase PA (uPA) plays a major role [32,33]. Interestingly, the uPA/plasmin system, in addition to acting directly on cartilage macromolecules, has been shown to be responsible for increased levels of other proteases, including collagenase [32,34]. The specific PAR-2 activation eliciting increased levels of MMP-1 and MMP-13 strongly suggests the likely involvement of this serine protease system in *in vivo *PAR-2 activation. Moreover, interaction between uPA and COX-2 was also shown in some cancer cells [35,36] and in corneal injury and inflammation [37].

Findings of previous studies have identified a role for PAR-2 in modulation of inflammation in rodent models, including inflammatory arthritis [11]. Here, we showed that, in addition to inflammatory factors such as COX-2, PAR-2 activation upregulates two MMPs, providing a critical link between inflammation and tissue destruction and thus contributing to the perpetuation of the altered responses of the chondrocytes. Interestingly, the predominant effect of PAR-2 activation over IL-1β on both MMPs and COX-2 reinforces the suggestion that PAR-2 is an upstream mediator of catabolic events [38,39]. Hence, data from this study suggest that PAR-2 activation would play a key role in the catabolic and inflammatory pathways that take place during OA by inducing the synthesis of major catabolic and inflammatory mediators via the p38 and p42/44 signalling pathways. Furthermore, PAR-2 upregulation by proinflammatory cytokines would amplify its effect.

## Conclusion

In summary, we showed, for the first time, that PAR-2 activation in OA cartilage participates in catabolic and inflammatory pathways induced during OA progression. Moreover, the present knowledge points to a possible therapeutic value for PAR-2 antagonists in the treatment of OA, not only as an anti-catabolic and anti-inflammatory but as an analgesic as well. Indeed, the proanalgesic properties of PAR-2 have shown that its activation plays a pivotal role in pain transmission with a direct effect on nociception and hyperalgesia [7,40-42]. This molecule, therefore, is believed to be an attractive target in OA because reducing its excess production may not only slow the disease progression, but also likely reduce the symptoms, enabling it to reach two targets simultaneously.

## Abbreviations

COX-2 = cyclooxygenase 2; C_T _= threshold cycle; DMEM = Dulbecco's modified Eagle's medium; Erk1/2 = extracellular signal-regulated kinase 1/2; FCS = fetal calf serum; GAPDH = glyceraldehydes-3-phosphate dehydrogenase; IL-1β = interleukin 1 beta; JNK = c-jun N-terminal kinase; MAP = mitogen-activated protein; MEK1/2 = mitogen-activated protein kinase kinase; MMP = matrix metalloproteinase; NF-κB = nuclear factor-kappa B; OA = osteoarthritis; PA = plasminogen activator; PAR = proteinase-activated receptor; PAR-2-AP = proteinase-activated receptor-2-activating peptide; PBS = phosphate-buffered saline; PCR = polymerase chain reaction; p-Erk1/2 = phosphorylated form of extracellular signal-regulated kinase 1/2; p-JNK = phosphorylated form of c-jun N-terminal kinase; p-p38 = phosphorylated form of p38; SD = standard deviation; SDS = sodium dodecyl sulfate; TGF-β1 = transforming growth factor-beta-1; TNF-α = tumor necrosis factor-alpha; TTBS 1× = Tris 20 mM, NaCl 150 mM (pH 7.5), and 0.1% Tween 20; uPA = urokinase plasminogen activator.

## Competing interests

The authors declare that they have no competing interests.

## Authors' contributions

CB participated in study design, acquisition of data, analysis and interpretation of data, statistical analysis, and manuscript preparation. NA participated in acquisition of data, analysis and interpretation of data, statistical analysis, and manuscript preparation. JMP and JPP participated in study design, analysis and interpretation of data, and manuscript preparation. ND participated in study design and in analysis and interpretation of data. HF participated in study design. All authors read and approved the final manuscript. CB and NA contributed equally to this work.
